# The expression of a tubby-like protein from *Malus domestica* (*Md*TLP7) enhances abiotic stress tolerance in *Arabidopsis*

**DOI:** 10.1186/s12870-019-1662-9

**Published:** 2019-02-06

**Authors:** Jianing Xu, Shanshan Xing, Qinghua Sun, Chunyan Zhan, Xin Liu, Shizhong Zhang, Xiaoyun Wang

**Affiliations:** College of Life Science, State Key Laboratory of Crop Biology, Shandong Agricultural University, Shandong Taian, 271018 People’s Republic of China

**Keywords:** Tubby-like protein, *Md*TLP7, Functional site, Stress tolerance, Subcellular localization

## Abstract

**Background:**

Tubby-like proteins (TLPs), characterized by a signature tubby domain, are widespread in plants and animals. To date, only plant TLPs involved in multifarious stress responses and male gametophyte development have been identified. However, studies on the molecular functions of plant TLPs are largely unknown.

**Results:**

In this investigation, the roles of a TLP from *Malus domestica* (*Md*TLP7) in response to abiotic stresses were characterized by expressing it in *Arabidopsis.* The expression of wild-type full-length *Md*TLP7 (FL) significantly increased the stress tolerance of *Arabidopsis* seedlings to osmotic, salt, cold and heat stress, while the expression of truncated *Md*TLP7 containing only the tubby domain (Tub) also showed some function. Located on a central α helix surrounded by 12 anti-parallel β strands in the tubby domain, the K_190_/R_192_ site may be involved in fixation to the plasma membrane, as shown by 3D homology modelling with animal TLPs. This site might play a crucial role in anti-stress functions since site-directed mutagenesis of *Md*TLP7 reduced stress tolerance. Subcellular localization showed that *Md*TLP7 was mainly localized in the plasma membrane in plant cells, suggesting that it might participate in the transduction of stress signals.

**Conclusions:**

The results of this study showed that *Md*TLP7 could improve abiotic stress tolerance not only in bacteria but also in plants. The K_190_/R_192_ residues in the tubby domain were not only the plasma membrane binding site of *Md*TLP7 but also played a key role in stress tolerance. These results may provide a basis for further exploring the mechanism of anti-stress functioning and downstream target genes of plant TLPs.

**Electronic supplementary material:**

The online version of this article (10.1186/s12870-019-1662-9) contains supplementary material, which is available to authorized users.

## Background

The tubby-like proteins (TLPs) are widely distributed in the animal and plant kingdoms. There are 5 members of the TLP family in mice, 4 in *Homo sapiens*, 9 in apple and 11 in *Arabidopsis* [1–4]. In mammals, many cellular functions of TLPs are involved in vesicular trafficking, the mediation of insulin signalling, gene transcription, G-protein signalling, and ribosomal RNA synthesis [5–9]. Although several *TLP*s in plants have been identified, their roles are elusive. In *Arabidopsis*, *At*TLP3 and *At*TLP9 are involved in ABA-dependent signalling during germination [3]. Several plant *TLP*s are upregulated under abiotic and biotic stress [4, 10–13].

Our previous study showed that a *TLP* gene from *Malus domestica* (*MdTLP7*) was upregulated in the transcriptional profile of apple under cold stress [14]. Heterologous expression of *MdTLP7* significantly increased the stress tolerance of *E. coli* cells against different abiotic stresses [15]. In contrast to animal TLPs, *Md*TLP7 contains a highly conserved F-box domain at its N-terminus in addition to the characteristic tubby domain [15]. In eukaryotes, F-box proteins participate in diverse cellular processes such as the response to stress, signal transduction, and the development of floral organs, primarily as a component of the Skp1-cullin-F-box (SCF) complex in protein ubiquitination [16–20].

Subcellular localization of TLPs clarifies their functions. Many mammalian and plant TLPs are usually localized in the plasma membrane and nucleus [12, 21]. As to the mechanism of TLP binding to the plasma membrane, a phosphatidylinositol-4,5-bisphosphate (PIP_2_) binding site involving two conserved residues in the tubby domain, lysine_330_ (K_330_) and arginine_332_ (R_332_), interacted directly with the membrane lipid of the plasma membrane, as demonstrated in two TLPs from mouse (TUB and TULP1) [22]. These two positively charged phosphate-coordinating residues (K_330_ and R_332_) are highly conserved not only in the animal TLPs but also in quite a few plant TLPs. The corresponding plasma membrane binding site in the tubby domain of *At*TLP3 (K_187_/R_189_) also localizes *At*TLP3 in the plasma membrane. Mutations of these conservative residues to other residues could disrupt the localization of *At*TLP3 to the plasma membrane [23]. PIP_2_ affects cell signalling by binding target proteins or enzymes [24]. When plants suffer abiotic stress-inducing agents such as mannitol, NaCl and H_2_O_2_ treatments, *At*TLP3 can trigger signals by detaching from the plasma membrane and moving through the cytosol to the nucleus to regulate the expression of genes [12, 23]. As bipartite transcription factors (TFs), mouse TLPs have DNA binding domains near their C-terminus and transcription modulation segments at their N-terminus [5]. TFs usually bind to *cis*-regulatory regions of a gene, which act as molecular switches controlling various biological processes including abiotic and biotic stress responses [25, 26]. A chickpea TLP (*Ca*TLP1) was demonstrated to be a putative TF presumably involved in multivariate stresses [11]. A rice TLP (*Os*TLP2) has been shown to bind to the promoter of *OsWRKY13* to regulate disease resistance [10]. These reports suggest that TLPs might participate in stress signal transduction as TFs.

To determine the functions of *Md*TLP7 in plants, full-length and truncated *Md*TLP7 (without the F-box domain) were transformed into *Arabidopsis* in this study. The responses of transgenic plants to different stresses were assessed. Using 3D structure modelling and site-directed mutation experiments, we found a critical site in the tubby domain of *Md*TLP7 for abiotic stress tolerance. These results open up new horizons for studying the mechanism of action of stress tolerance in plant TLPs and may promote the development of methods to improve plant stress resistance.

## Results

### Expression of *MdTLP7* enhanced abiotic stress tolerance in *Arabidopsis*

To examine the function of *MdTLP7* in the plant stress response, the full-length cDNAs of wild-type full-length *MdTLP7* (FL) and truncated *MdTLP7* with only the tubby domain (Tub) were transformed into wild-type *Arabidopsis* (WT) by the CaMV 35S promoter. More than 10 transgenic lines were identified by kanamycin testing and PCR amplification (T1 generation, Additional file 1: Figure S1). Homozygous lines were identified by screening for non-segregation from each independent transformant (T3 generation). The 2 homozygous FL transgenic lines (named FL-1, FL-2) and 2 Tub transgenic lines (named Tub-1, Tub-2) with high expression were chosen for further analysis (Additional file 2: Figure S2).

**Fig. 1:**
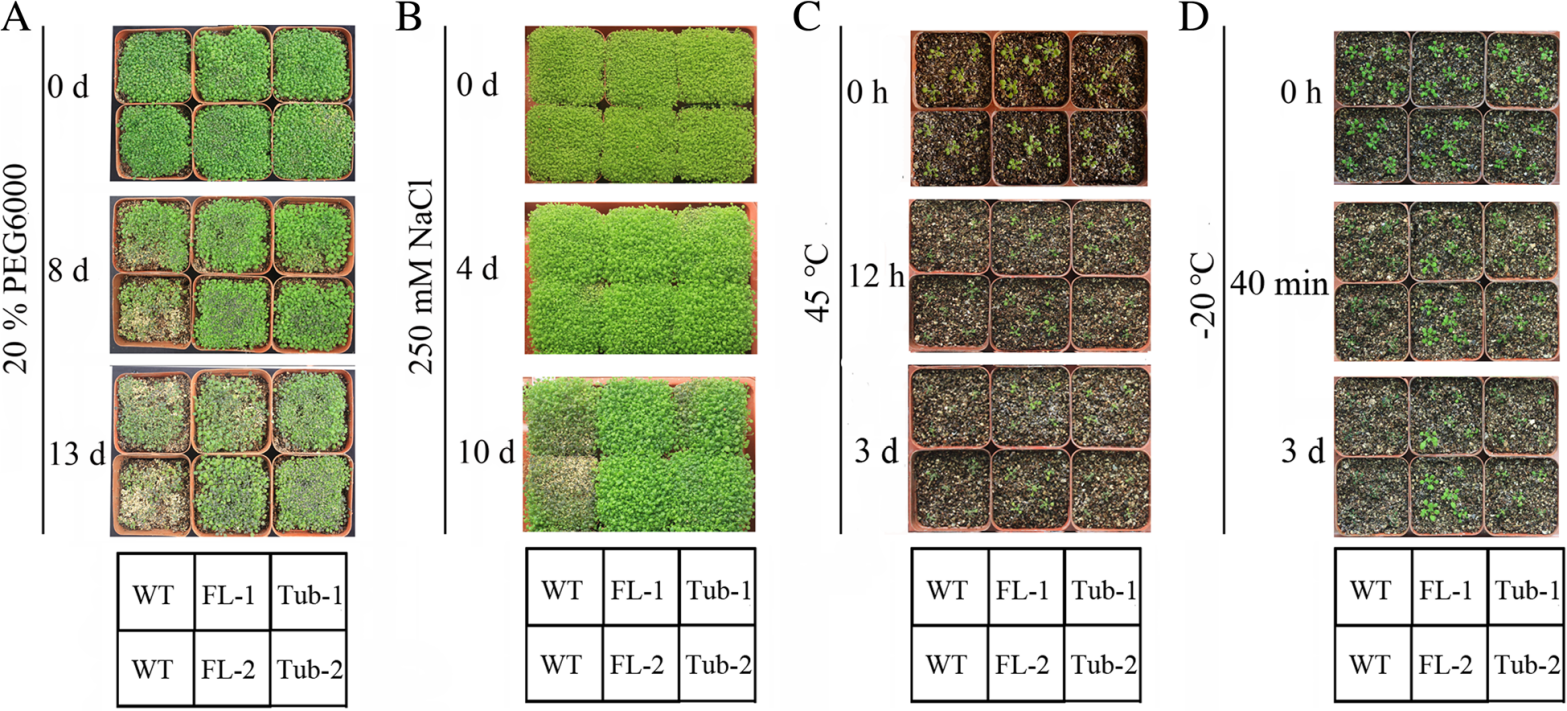
Phenotypes of *MdTLP7* transgenic plants under abiotic stress. **a** 20% PEG6000 treatment for 13 days, **b** 250 mM NaCl treatment for 10 days, **c** 45 °C treatment for 12 h and then normal growth conditions (22 °C) for 3 days, and **d** successive pre-treatments at 5 °C to − 5 °C for 10 h followed by treatment at − 20 °C for 40 min and normal growth conditions (22 °C) for 3 days

Under PEG and salt treatments, the growth of both transgenic lines, FL and Tub, was better than that of WT; little difference was found between the FL and Tub lines (Fig. 1a and b). The results suggested that the expression of wild-type *Md*TLP7 (FL) or truncated *Md*TLP7 (Tub) enhanced tolerance to osmotic and salt stresses.

To induce temperature stress, we placed seedlings in hot (45 °C) and cold (− 20 °C) environments. After treatment at 45 °C for 12 h, obvious differences were observed among the transgenic lines (FL and Tub) and between the transgenic lines and WT *Arabidopsis,* and the growth of the Tub plants was better than that of the WT plants but worse than that of the FL plants. After 3 days of recovery, all of the WT plants were dead whereas 80% of the FL transgenic plants and 40% of the Tub transgenic plants survived (Fig. 1c). Similar results after cold treatment were observed among the WT, FL and Tub lines. The seedlings were pre-treated at 5°Cto − 5 °C for 10 h and then subjected to − 20 °C for 40 min. All of the WT plants were dead whereas 70% of the FL plants and 30% of the Tub plants survived after 3 days of recovery (Fig. 1d).

The results indicated that the expression of *Md*TLP7 in *Arabidopsis* could enhance tolerance to several abiotic stressors in *Arabidopsis*. Truncated *Md*TLP7 (Tub) still retained almost complete function compared to that of wild-type *Md*TLP7 (FL) under both PEG and salt stress. Regarding temperature stress, truncated *Md*TLP7 (Tub) only had approximately half or less of the anti-stress function of wild-type *Md*TLP7 (FL). The results suggested that the tubby domain of *Md*TLP7 has key roles and that the F-box domain may have synergistic effects with the tubby domain in the stress response.

### *Md*TLP7 is localized in the plasma membrane

**Fig. 2:**
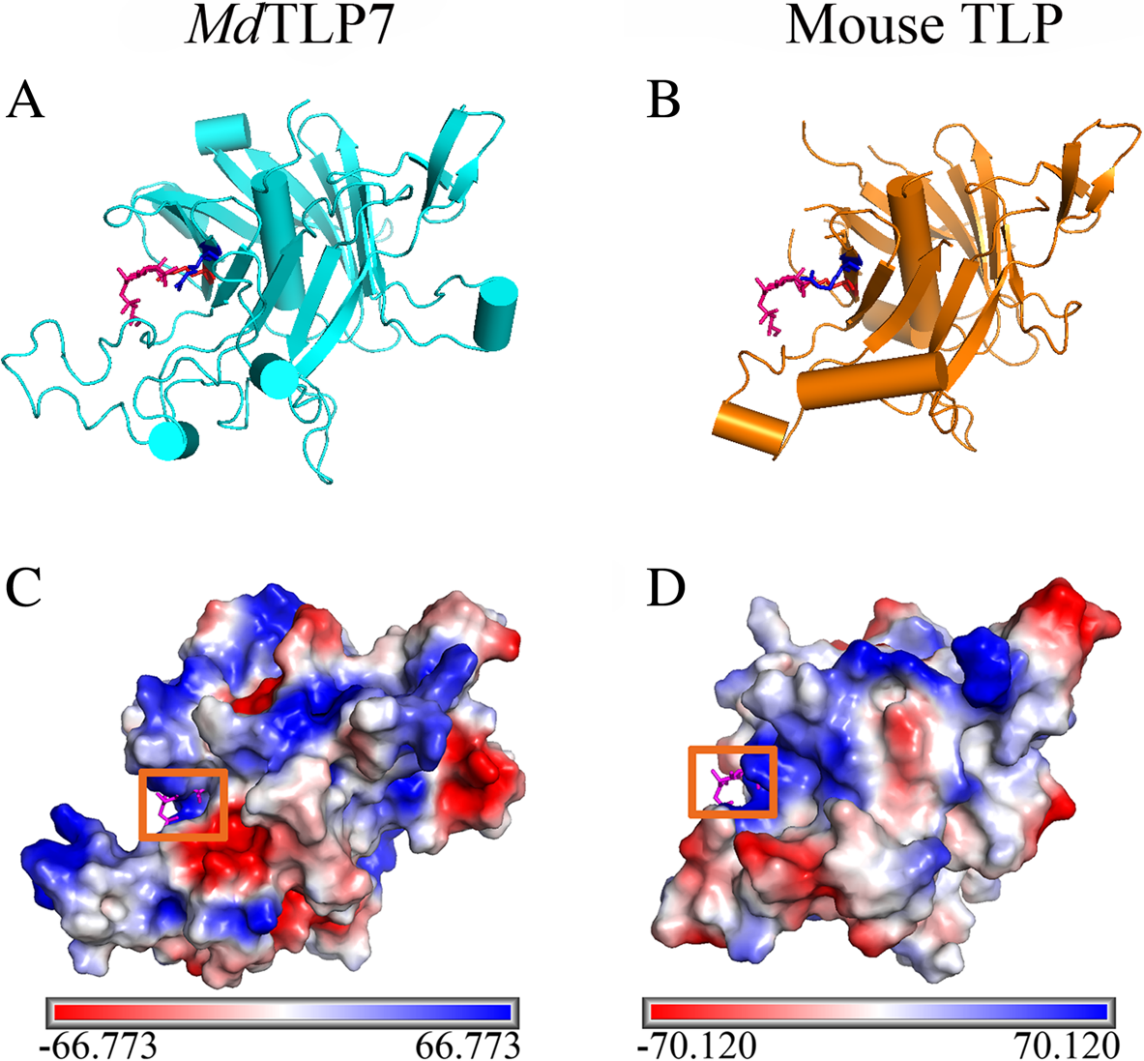
3D structures of the tubby domain of *Md*TLP7 and mouse tubby protein. **a**-**b** 3D homology model of *Md*TLP7 (cyan) and mouse tubby protein (PDB identifier 1I7E; orange) showing K_190_ (red) and R_192_ (blue). IBS (pink), an analogue of PIP2, was used to show the conserved binding site. **c**-**d** Electrostatic surface of the tubby domain generated with the programme PyMOL. Electrostatic surface of *Md*TLP7 with IBS modelled (**c**, orange frame) and mouse tubby protein-bound IBS (**d**, orange frame). The positive charges, negative charges and neutral charges are blue, red and white, respectively

A 3D homology model of the tubby domains of *Md*TLP7 was established using SWISS-MODEL that is highly consistent with the mouse TLP (TULP3) structure. Both contain a central α helix and a closed 12-stranded β barrel (Fig. 2a and b). Mouse TLPs are tethered to the plasma membrane via a PIP_2_ binding site (K_330_/R_332_) in the tubby domain [8]. The corresponding amino acid residues in *Md*TLP7, K_190_/R_192_, show high positional overlap with residues K_330_/R_332_ of mouse TLP, the TLP plasma membrane binding site. Both grooves of the plasma membrane binding site in *Md*TLP7 and mouse TLP were positively charged as shown by electrostatic surface analysis (Fig. 2c and d). The results suggested that *Md*TLP7 may also localize in the plasma membrane of plant cells, similar to mouse TLP in animal cells.

**Fig. 3:**
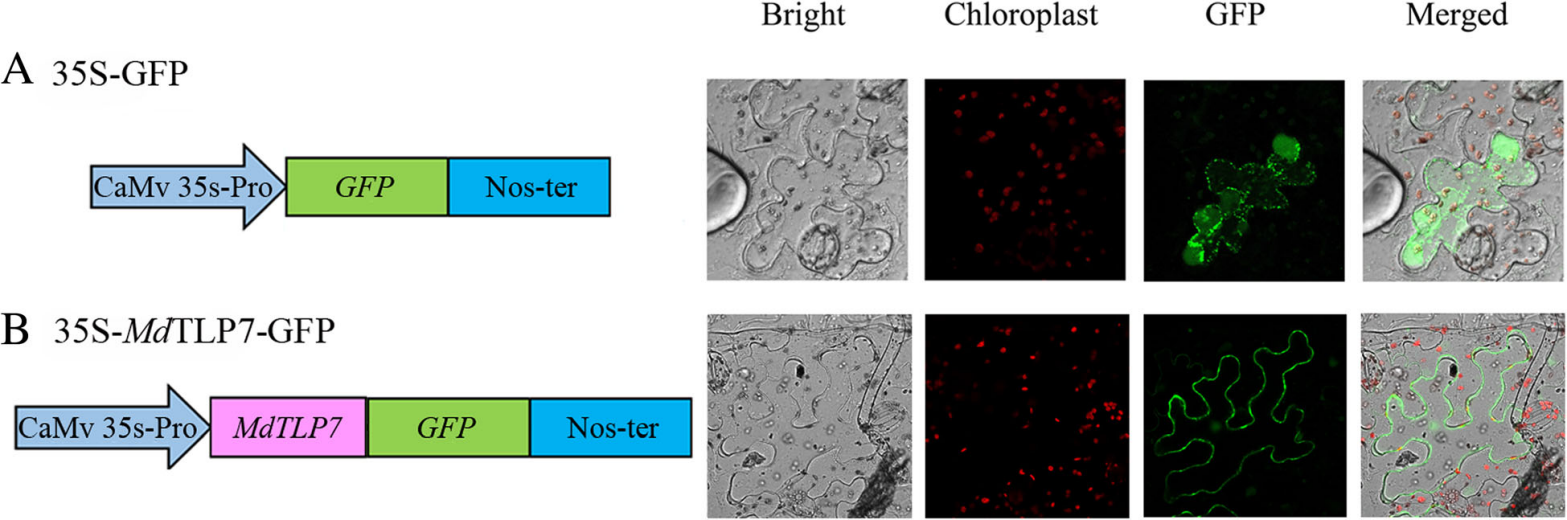
*Md*TLP7 was localized in the PM of tobacco cells by the PIP_2_ binding site. **a** The subcellular location of 35S-GFP protein. **b** The subcellular location of 35S-*M*dTLP7-GFP protein. Green fluorescence indicates the location of GFP. Red fluorescence indicates the location of the chloroplast

To investigate whether *Md*TLP7 localized in the plasma membrane, the sequence encoding *Md*TLP7 was fused to the sequence of GFP. As shown in Fig. 3, the GFP signal emitted by the fusion protein was localized in the plasma membrane of tobacco leaf cells, while the GFP signal of the empty vector was localized in the cytoplasm and plasma membrane.

### K_190_/R_192_ in the tubby domain played crucial roles in stress tolerance

A PIP_2_ analogue, L-α-glycerophospho-D-myoinositol-4,5-bisphosphate (IBS), was used to check changes in the charge of the corresponding plasma membrane binding site of *Md*TLP7. When K_190_/R_192_ were mutated to alanine residues, the electrostatic surface of the IBS-bound groove changed from positive to neutral charge, although the whole tertiary structure of the tubby domain was not significantly affected in 3D homology modelling (Additional file 3: Figure S3 and Additional file 4: Figure S4). Whether the positive charge of the active groove is indispensable for the function of *Md*TLP7 needs to be identified.

To investigate the effects of this site on the anti-stress response, point mutants of *Md*TLP (K_190_A, R_192_A or K_190_A/R_192_A) were constructed and transformed into *E. coli*. A survival test on solid medium showed that the survival ratio of cells expressing wild-type *Md*TLP7 was significantly higher than that of *Md*TLP mutants under salt stress conditions (Additional file 5: Figure S5).

**Fig. 4:**
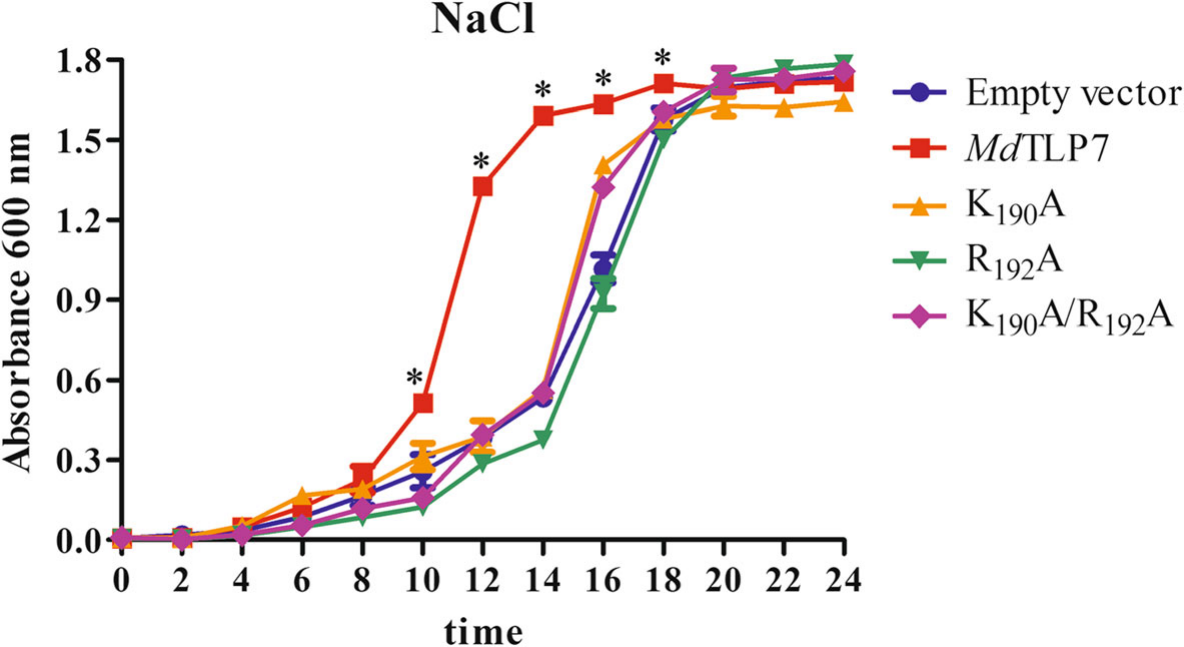
Growth analysis of *E. coli* cells expressing full length *Md*TLP7 and three *Md*TLP7 point mutants. *E. coli* cells were cultivated in LB medium supplemented with 500 mM NaCl. OD600 was recorded at 2 h intervals for 24 h, and the mean values are represented in the graph. *p < 0.001

The growth curves of wild-type *Md*TLP7 and *Md*TLP7 mutants in 500 mM NaCl-containing liquid medium were assayed to further ascertain the site conferring stress resistance to *Md*TLP7. The cells expressing wild-type *Md*TLP7 entered the exponential growth stage after 10 h of cultivation, which was much earlier than the cells expressing mutant *Md*TLP7 (K_190_A, R_192_A or K_190_A/R_192_A). The three mutants entered the exponential growth stage at approximately 15 h, which was quite similar to the time frame of the empty vector (Table 1 and Fig. 4). These results revealed that the plasma membrane biding site at K_190_/R_192_ of *Md*TLP7 played a key role in abiotic stress tolerance.

**Table 1:** Nonlinear regression analysis of *E. coli* cell lines growth curve under salt stress

Sample	Equation	*r* ^2^	Inflection point of time (h)
Empty vector	Y = 1.830/(1 + 1066e^-0.458t^)	0.987	15.2
*Md*TLP7	Y = 1.703/(1 + 7956e^-0.837t^)	0.995	10.7
K_190_A	Y = 1.700/(1 + 2111e^-0.539t^)	0.970	14.2
R_192_A	Y = 1.834/(1 + 13032e^-0.598t^)	0.994	15.8
K_190_A/R_192_A	Y = 1.783/(1 + 6736e^-0.602t^)	0.991	14.6

## Discussion

The tubby domain is the signature conserved domain of the tubby-like family [9]. In addition to the tubby domain, almost all of the plant TLPs have an F-box domain at their N-terminal regions [21]. *Md*TLP7 has two functional domains: an F-box domain at the N-terminus and a tubby domain at the C-terminus [4]. Some F-box domain-containing proteins are involved in the stress response by interacting with S-phase kinase association protein 1 to form an SCF complex and participating in the ubiquitin-proteasome system [27]. In our previous study, the F-box domain of *Md*TLP7 was not essential for the anti-stress function of bacteria since truncation of the F-box did not significantly affect its anti-stress resistance [15]. In this study, only expressing the Tub domain of *Md*TLP7 in *Arabidopsis* significantly increased tolerance to salt and PEG stress. Regarding cold and heat stress, the expression of full-length *Md*TLP7 was much better for tolerating cold and heat stress than the expression of truncated *Md*TLP7. The presence of the conserved F-box domain in *Md*TLP7 might play a role in stress tolerance through the ubiquitin-proteasome pathway, which is a major posttranscriptional regulatory process related to redundant proteins in eukaryotes.

Boggon et al. determined that mouse TLPs were localized in the plasma membrane via a plasma membrane binding site (K_330_/R_332_) in a structure of the tubby domain bound to PIP_2_ [5]. The 3D structure showed high positional overlap between K_190_/R_192_ in *Md*TLP7 and K_330_/R_332_ in mouse TLP (Fig. 2). Therefore, *Md*TLP7 might also localize in the plasma membrane via K_190_/R_192_ binding to PIP_2_. PIP_2_ is a kind of membrane phospholipid that affects cell signalling by binding target proteins to generate bioactive inositol phosphates [24]. In this investigation, we found that *Md*TLP7 localized in the plasma membrane of tobacco cells, suggesting that it might participate in stress signal transduction (Fig. 3). However, studies on the relationship between the plasma membrane binding site and the protein functions of plant TLP have not been reported. The survival ratios of cells expressing *Md*TLP7 point mutants (K_190_A and R_192_A) were slightly higher than that of cells expressing the empty vector under stress. However, the survival ratio of cells expressing *Md*TLP7 was significantly higher than that of cells expressing *Md*TLP7 mutants under salt stress condition (Additional file 5: Figure S5). The growth curve assay also showed that the cells expressing wild-type *Md*TLP7 entered the exponential growth stage much earlier than the cells expressing mutant *Md*TLP7 (Table 1 and Fig. 4). The survival tests on solid and liquid medium both revealed that the plasma membrane binding site at K_190_/R_192_ of *Md*TLP7 played a key role in abiotic stress tolerance. The 3D homology model and electrostatic surface analysis of *Md*TLP7 showed that point mutations at K_190_/R_192_ did not change the 3D structure of *Md*TLP7, but the PIP_2_ binding groove changed from positively charged to neutral, indicating that the positive charge of the groove was important to the anti-stress function of *Md*TLP7 (Additional file 3: Figure S3 and Additional file 4: Figure S4).

## Conclusions

The results of this study showed that *Md*TLP7 could improve abiotic stress tolerance not only in bacteria but also in *Arabidopsis*. The expression of full-length wild-type *Md*TLP7 significantly increased the stress tolerance of *Arabidopsis* seedlings to osmotic, salt, cold and heat stress, while the expression of truncated *Md*TLP7 including only the tubby domain also retained some function. The K_190_/R_192_ residues of *Md*TLP7 were found to be key amino acids involved in stress tolerance for the first time. In the future, we will further study the molecular mechanism of *Md*TLP7 in abiotic stress tolerance.

## Methods

### Expression of *MdTLP7* in transgenic plants

In this study, *Arabidopsis thaliana* ecotype Columbia-0 (Col-0) was used as the experimental material. *MdTLP7* cDNA containing the complete open reading frame (ORF) and cDNA containing the tubby domain of *MdTLP7* were amplified from the *MdTLP7*-pET30a recombinant vector, which was constructed in our previous study [15]. The full-length *MdTLP7* (FL) and tubby domain (Tub) cDNAs were inserted into the pBI121 vector, resulting in two recombinant vectors (pBI121-FL, pBI121-Tub). The pBI121-FL and pBI121-Tub recombinant vectors were integrated into *Arabidopsis* Columbia-0 by *Agrobacterium tumefaciens* (GV3101)-mediated transformation. The floral dip method was used for this transformation [28]. Transformants (T1) were selected on Murashige and Skoog medium agar plates (50 μg/mL kanamycin). The presence and integrity of the FL or Tub cDNAs in transformants (T1) were further confirmed by PCR amplification. The primer sequences used in this experiment are listed in Additional file 6: Table S1. The seeds from the putative transgenic plants were again selfed to obtain T3 progenies through T2 plants. The transgenic (T3 generation) and WT *Arabidopsis* seeds were used for further experiments.

Quantitative real-time PCR (qRT-PCR) was performed to investigate the expression of FL and Tub in *Arabidopsis*. Two FL transgenic plants (named FL-1 and FL-2), Tub transgenic plants (named Tub-1 and Tub-2) and wild-type (WT) *Arabidopsis* plants were randomly selected. FL-1 was used as a control, and the *actin* gene was used as an internal control. The primer sequences used in this experiment are listed in Additional file 6: Table S1. The experiments were carried out at least three times under identical conditions. The relative expression levels were calculated using the 2^-(ΔΔCt)^ method [29].

### Plant growth conditions and stress treatments

The WT, FL and Tub plants were grown in vermiculite at a constant temperature of 22 °C under 8 h illumination at 120 μmol m^− 2^ s^− 1^ and a 16 h dark cycle. WT, FL and Tub plants were grown to 10 days of age. To induce osmotic stress, 10-day-old seedlings were irrigated with a 20% PEG6000 solution every day for up to 13 days. To induce salt stress, 10-day-old seedlings were irrigated with a 250 mM NaCl solution every day for up to 10 days.

Forty *Arabidopsis* plants from four independent lines grown for 10 days at 22 °C were subjected to temperature stress treatment. To induce heat stress, 10-day-old seedlings were grown at 45 °C for 12 h and then returned to a growth chamber at 22 °C under 8 h illumination at 120 μmol m^− 2^ s^− 1^ and a 16 h dark cycle for 3 days. To induce cold stress, 10-day-old seedlings were pre-treated at 5 °C to − 5 °C for 10 h successively and then subjected to − 20 °C for 40 min. After cold treatment, the seedlings were returned to a growth chamber at 22 °C under 8 h illumination at 120 μmol m^− 2^ s^− 1^ and a 16 h dark cycle for 3 days before scoring the survival of the seedlings.

### Subcellular localization of *Md*TLP7

To determine the subcellular localization of *Md*TLP7, the *MdTLP7* ORF without a termination codon was inserted upstream of the GFP gene. The 35S-GFP plasmid was used as a control. The subcellular localization experiment was carried out by *Agrobacterium tumefaciens* infiltration into the leaves of tobacco as described by Jia et al. [30]. After 48 h of infiltration, a two photon laser confocal microscope (ZEISS, Germany) was used to observe the fluorescence in tobacco cells. Fluorescence was detected using a 505 to 550 nm bandpass filter for GFP. Image processing was performed with the Zeiss LSM image processing software (Zeiss).

### Construction of *MdTLP7* site-directed mutants

Three point mutations (K_190_A, R_192_A and K_190_A/R_192_A) were made by a QuikChange™ mutagenesis kit using the mutant primers listed in Additional file 6: Table S1. The three *MdTLP7* point mutants were all inserted into the pET30a (+) expression plasmid (Novagen) and transformed into *E. coli* cells. All the constructed plasmids were sequenced by Sunny Biotechnology Company (Shanghai China) to confirm the correct sequences.

### Survival test under salt stress on solid medium and in solution medium

The survival test and growth analysis under salt stress of the *E. coli* described above were carried out as described by Du et al. [15]. The cells were grown in LB solution medium to an OD600 of 0.4–0.6 at 37 °C, and expression of the recombinant proteins was induced for 2 h with 0.5 mM isopropyl-β-D-thiogalactopyranoside at 37 °C. For the survival test on solid medium, cultures were diluted to an OD600 of 0.6 and then diluted to 10^− 2^, 10^− 3^ and 10^− 4^. Ten microliter cultures from each dilution (10^− 2^, 10^− 3^ and 10^− 4^) were plated on solid LB medium supplemented with 500 mM NaCl and then incubated at 37 °C for 16 h. The colony number on each plate for the culture diluted to 10^− 3^ was counted after incubation. Each experiment was repeated 3 times, and the data are presented as the mean with error bars indicating the standard deviation. The significance of the differences between groups was estimated using Student’s *t*-test. A value of *P* < 0.01 indicated a significant difference.

For the survival test in solution medium, cultures were diluted to an OD600 of 0.6 and 20 μl of the cultures were used to inoculate 10 ml LB solution containing a high concentration of 500 mM NaCl before incubation at 37 °C on a rotary shaker (150 rpm). The bacterial suspension was harvested every 2 h until 24 h had passed, and the OD600 was measured. Each experiment was repeated 3 times, and the data are presented as the mean with error bars indicating the standard deviation. All statistical analyses were performed using SPSS 19.0 (IBM, SPSS, Chicago, IL). Figures were made by GraphPad Prism 5 (GraphPad Software Inc., San Diego, CA, USA). Nonlinear regression analysis was fit using a logistic model (Y = A/ (1 + Be^-kt^)). The inflection point of the time ((lnB)/k) of each sample was calculated. The significance of the differences between groups was estimated using Student’s *t*-test. A value of *P* < 0.001 indicated a significant difference.

### 3D structure modelling

The 3D structural models of *Md*TLP7 and *Md*TLP7 mutants were constructed using the protein structure homology-modelling server SWISS-MODEL [31–33]. The most suitable template structure searched by the SWISS-MODEL server was used. To compare the spatial position of *Md*TLP7 bound to PIP_2_, a mouse brain tubby protein-bound PIP_2_ structure (PDB identifier 1I7E) was used as a 3D model. Visualization of the protein molecules and vacuum electrostatics analysis was performed using the PyMOL Molecular Graphics System (Version 1.7.4.5, https://pymol.org/2/, Schrödinger, LLC).

## Additional files

Additional file 1: **Figure S1.** PCR amplification of Kan-resistant seedlings. (1–10) PCR products of OE_FL_ transgenic lines, (11–20) PCR products of OE_Tub_ transgenic lines, M, DL2000 marker. (TIF 502 kb)

Additional file 2: **Figure S2.** qRT-PCR analysis of the expression of *MdTLP7* in the leaves of WT and transgenic plants. (TIF 1457 kb)

Additional file 3: **Figure S3.** Homology model of the Tubby domain of *Md*TLP7 and three *Md*TLP7 point mutants. (A) Tubby domain of *Md*TLP7. (B-D) Three *Md*TLP7 point mutants. K_190_ is shown in red, R_192_ in blue, and mutated amino acids in orange. (TIF 1046 kb)

Additional file 4: **Figure S4.** Electrostatic surface of the tubby domain of *Md*TLP7 and three *Md*TLP7 point mutations. (A) Electrostatic surface of the *Md*TLP7 tubby protein. (B-D) Electrostatic surfaces of the point mutants. Blue indicates a positive charge, red indicates a negative charge and white indicates a neutral charge. The groove of the IBS-bound region is shown in the green frame. (TIF 2105 kb)

Additional file 5: **Figure S5.** Survival test of *E. coli* expressing *Md*TLP7, empty vector or point mutants of under salt stress. (A) 10 μl cultures from 10^− 2^ to 10^− 4^ dilutions were spotted on LB plates treated with 0.5 M NaCl. (B) The colony numbers for the 10^− 3^ dilutions appearing on each plate were counted. *p < 0.01. (TIF 13567 kb)

Additional file 6: **Table S1.** Primers used in this experiment. (DOCX 12 kb)

## Abbreviations

3D: three-dimensional
A: alanine
FL: full-length of *MdTLP7*
IBS: L-α-glycerophospho-D-myoinositol 4,5-bisphosphate
K: lysine
ORF: open reading frame
PIP_2_: phosphatidylinositol-4,5-bisphosphate
qRT-PCR: Quantitative real-time PCR
R: arginine
SCF: Skp1-cullin-F-box
TF: transcription factor
TLP: tubby-like protein
Tub: tubby domain
WT: wild-type of *Arabidopsis*
