# Supplementary figures and images for: The expression of a tubby-like protein from Malus domestica (MdTLP7) enhances abiotic stress tolerance in Arabidopsis
Source: BMC Plant Biol. 2019 Feb 6;19:60. doi: 10.1186/s12870-019-1662-9 (PMC6366083; doi:10.1186/s12870-019-1662-9)

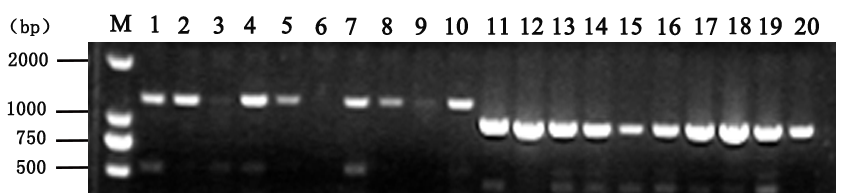

Supplement: Supplementary file 1 — Figure S1. PCR amplification of Kan-resistant seedlings. (1–10) PCR products of OEFL transgenic lines, (11–20) PCR products of OETub transgenic lines, M, DL2000 marker. (TIF 502 kb) [file 12870_2019_1662_MOESM1_ESM.tif]

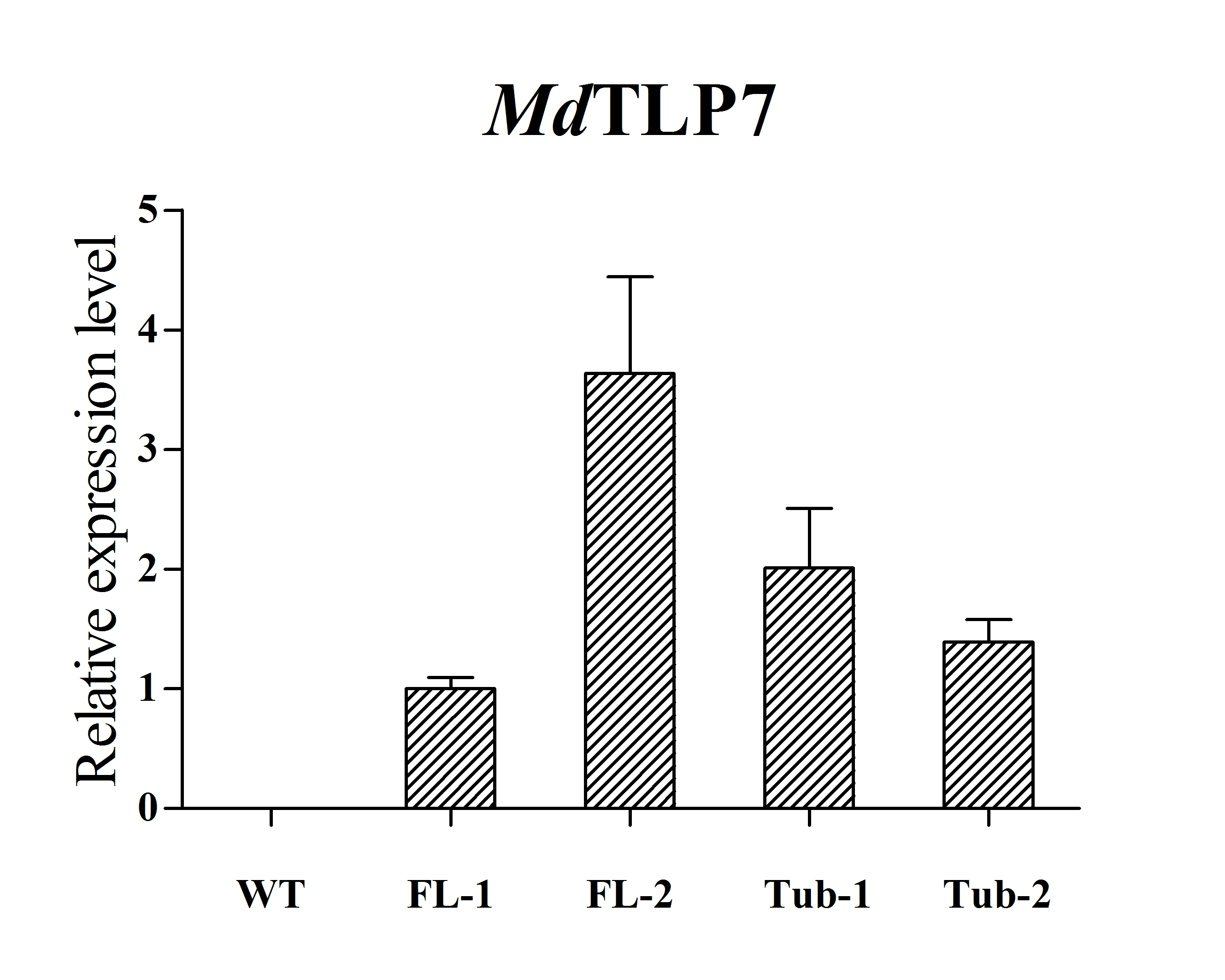

Supplement: Supplementary file 2 — Figure S2. qRT-PCR analysis of the expression of MdTLP7 in the leaves of WT and transgenic plants. (TIF 1457 kb) [file 12870_2019_1662_MOESM2_ESM.tif]

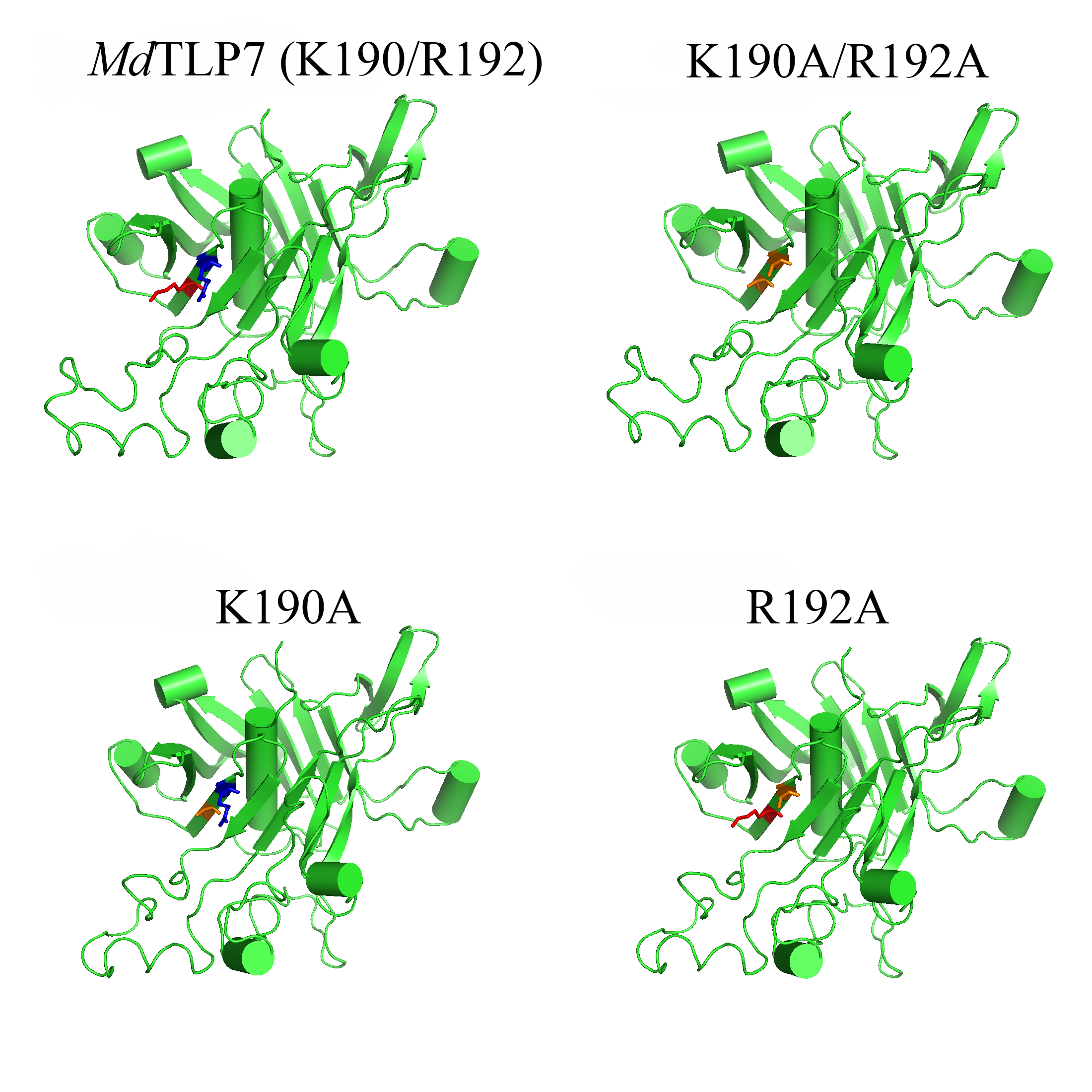

Supplement: Supplementary file 3 — Figure S3. Homology model of the Tubby domain of MdTLP7 and three MdTLP7 point mutants. (A) Tubby domain of MdTLP7. (B-D) Three MdTLP7 point mutants. K190 is shown in red, R192 in blue, and mutated amino acids in orange. (TIF 1046 kb) [file 12870_2019_1662_MOESM3_ESM.tif]

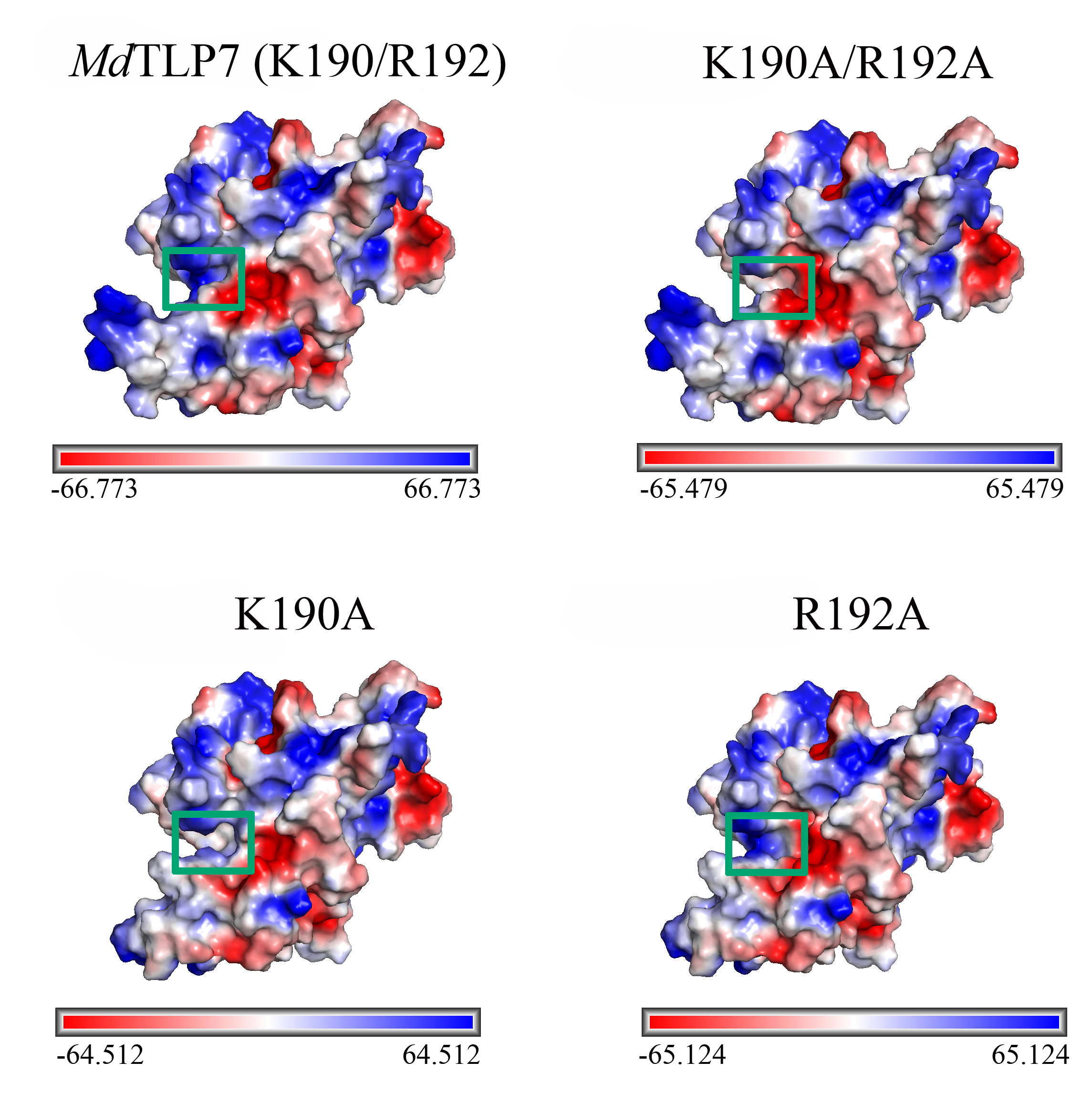

Supplement: Supplementary file 4 — Figure S4. Electrostatic surface of the tubby domain of MdTLP7 and three MdTLP7 point mutations. (A) Electrostatic surface of the MdTLP7 tubby protein. (B-D) Electrostatic surfaces of the point mutants. Blue indicates a positive charge, red indicates a negative charge and white indicates a neutral charge. The groove of the IBS-bound region is shown in the green frame. (TIF 2105 kb) [file 12870_2019_1662_MOESM4_ESM.tif]

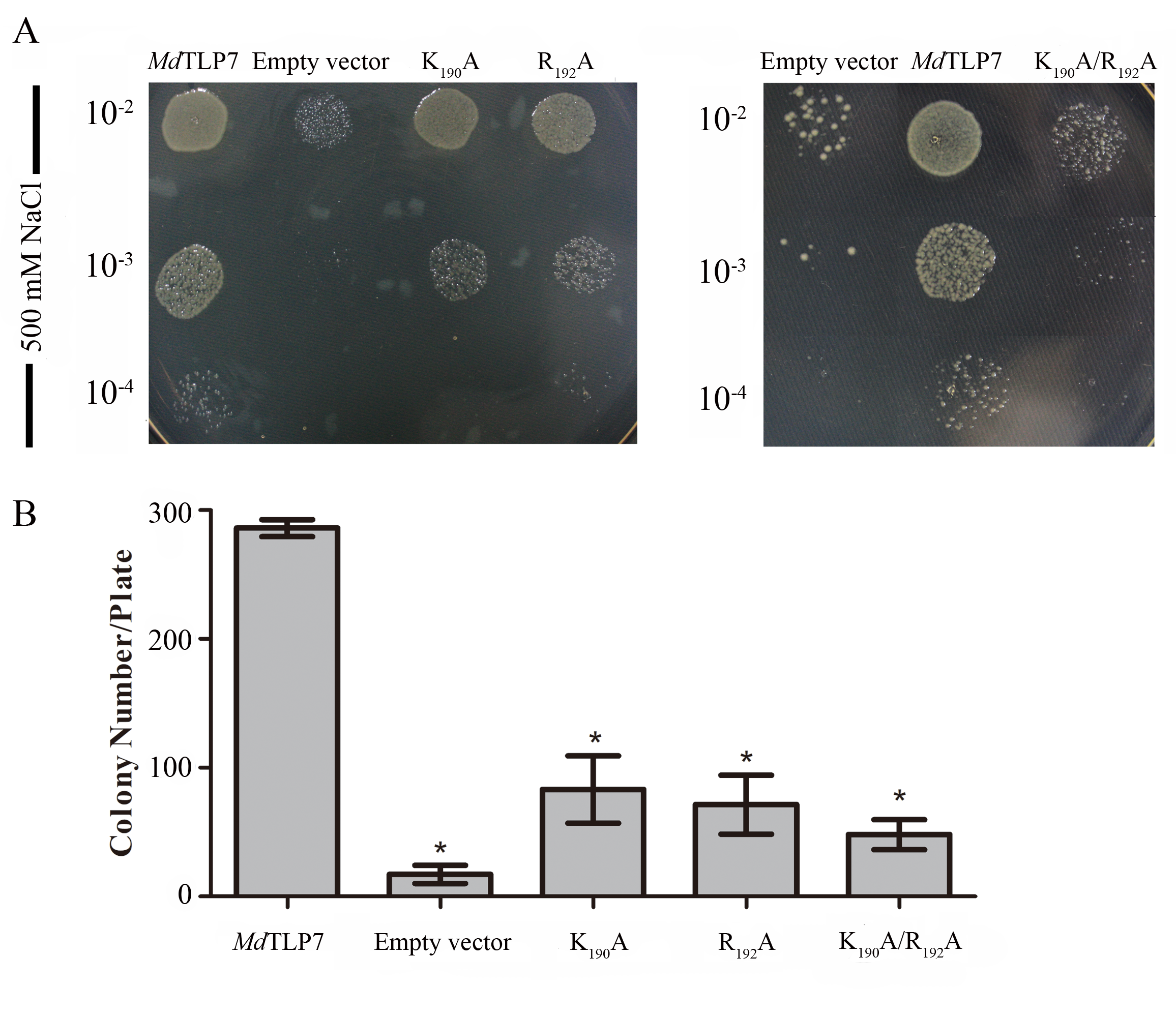

Supplement: Supplementary file 5 — Figure S5. Survival test of E. coli expressing MdTLP7, empty vector or point mutants of under salt stress. (A) 10 μl cultures from 10− 2 to 10− 4 dilutions were spotted on LB plates treated with 0.5 M NaCl. (B) The colony numbers for the 10− 3 dilutions appearing on each plate were counted. *p < 0.01. (TIF 13567 kb) [file 12870_2019_1662_MOESM5_ESM.tif]
