# Supplementary material for: The expression of a tubby-like protein from Malus domestica (MdTLP7) enhances abiotic stress tolerance in Arabidopsis
Source: BMC Plant Biol. 2019 Feb 6;19:60. doi: 10.1186/s12870-019-1662-9 (PMC6366083; doi:10.1186/s12870-019-1662-9)
Supplement: Supplementary file 6 — Table S1. Primers used in this experiment. (DOCX 12 kb) [file 12870_2019_1662_MOESM6_ESM.docx]

Table S1 Primers used in this experiment

| Primer Name | Sequence 5′-3′ |
| --- | --- |
| *MdTLP7*-F | GCGTCTAGAATGTCGTTCAAAGGC |
| *MdTLP7*-R | AATCCCGGGTCATTCACAAGCTG |
| *Tubby domian*-F | GGGTCTAGAATGAGGGATATAGTCA |
| *Tubby domian*-R | AATCCCGGGTCATTCACAAGC |
| qRT-F | TACAATGCTGGTGCCTTAACT |
| qRT-R | GGATTACCCTCTCTTGCTCTTC |
| *At*Actin-F | GGATGAACCAGAAGGATG |
| *At*Actin-R | CTCGGTAAGAAGAACAGG |
| *Md*TLP7_K190A_-F | GCACTACATATGTTGGTGCATTGAGGTCCAACT |
| *Md*TLP7_K190A_-R | GCACCAACATATGTAGTGCTGGCTCGAGAA |
| *Md*TLP7_R192A_-F | CACTACATATGTTGGTAAATTGGCGTCCAACTTTTTGG |
| *Md*TLP7_R192A_-R | GCCAATTTACCAACATATGTAGTGCTGGCTCGAGA |
| *Md*TLP7_K190A/R192A_-F | GCACTACATATGTTGGTGCATTGGCGTCCAACTTTTTGG |
| *Md*TLP7_K190A/R192A_-R | GCCAATGCACCAACATATGTAGTGCTGGCTCGAGAA |
